# Supplementary material for: Splice-Junction-Based Mapping of Alternative Isoforms in the Human Proteome
Source: Cell Rep. Author manuscript; Available in PMC 2020 Jan 15. (PMC6961840; doi:10.1016/j.celrep.2019.11.026)
Supplement: 2 [file NIHMS1546469-supplement-2.zip › DF1/PXD006675/AtrialSeptum/AtrialSeptum_20_TTN_ETPPVEVPGGEK.pdf]

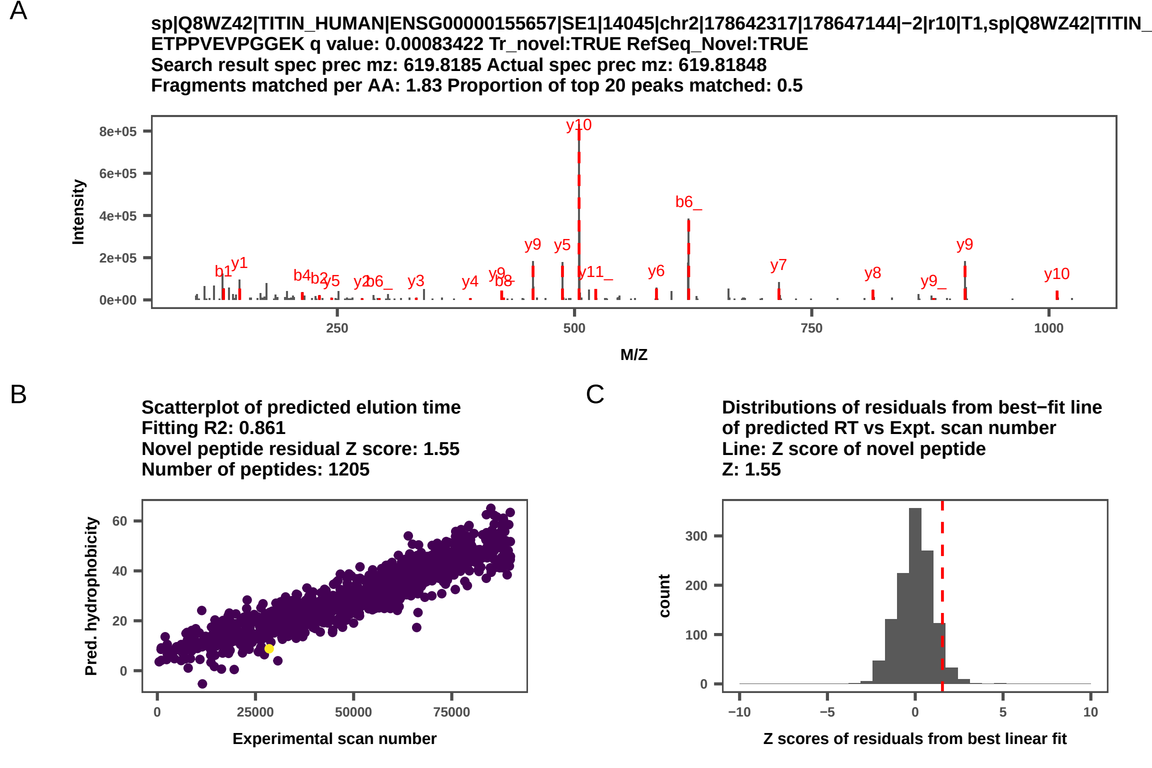

A
sp|Q8WZ42|TITIN_HUMAN|ENSG00000155657|SE1|14045|chr2|178642317|178647144|−2|r10|T1,sp|Q8WZ42|TITIN_
ETPPVEVPGGEK q value: 0.00083422 Tr_novel:TRUE RefSeq_Novel:TRUE
Search result spec prec mz: 619.8185 Actual spec prec mz: 619.81848
Fragments matched per AA: 1.83 Proportion of top 20 peaks matched: 0.5
Intensity
8e+05
6e+05
4e+05
2e+05
0e+00
y10
b6_
y9
y5
y9
y7
y11_
y6
y8
y10
b1
y1
b4
b2
y5
y2
b6_
y3
y4
y9_
b8
y9_
250
500
750
1000
M/Z
B
Scatterplot of predicted elution time
Fitting R2: 0.861
Novel peptide residual Z score: 1.55
Number of peptides: 1205
Pred. hydrophobicity
60
40
20
0
0
25000
50000
75000
Experimental scan number
C
Distributions of residuals from best−fit line
of predicted RT vs Expt. scan number
Line: Z score of novel peptide
Z: 1.55
count
300
200
100
0
−10
−5
0
5
10
Z scores of residuals from best linear fit
